# Associations between Vitamin D Status and Polysomnographic Parameters in Adults with Obstructive Sleep Apnea

**DOI:** 10.3390/life14020275

**Published:** 2024-02-18

**Authors:** Ioanna Kechribari, Meropi D. Kontogianni, Michael Georgoulis, Kallirroi Lamprou, Eleni Perraki, Emmanouil Vagiakis, Nikos Yiannakouris

**Affiliations:** 1 Department of Nutrition and Dietetics, School of Health Sciences and Education, Harokopio University of Athens, 17676 Athens, Greece; 2 Center of Sleep Disorders, 1st Department of Critical Care, Evangelismos General Hospital, 10676 Athens, Greece

**Keywords:** obstructive sleep apnea, apnea-hypopnea index, 25-hydroxyvitamin D, vitamin D deficiency

## Abstract

Vitamin D deficiency (VDD) may be associated with obstructive sleep apnea (OSA) presence and is more pronounced with increasing OSA severity; however, the relationship between these two entities remains unclear. This was a cross-sectional study among 262 adults with in-hospital-attended polysomnography-diagnosed OSA and no additional major comorbidities, aiming to explore possible associations between serum 25-hydroxyvitamin D [25(OH)D] levels and polysomnographic parameters. Data on demographics, medical history, anthropometric indices, and lifestyle habits were collected at enrolment. Serum 25(OH)D was evaluated using chemiluminescence, with VDD defined as 25(OH)D < 20 ng/mL. VDD was observed in 63% of the participants. Serum 25(OH)D correlated negatively with apnea–hypopnea index and other polysomnographic indices (all *p* < 0.05). In logistic regression analysis, adjusting for age, sex, smoking, body mass index, physical activity, dietary vitamin D intake, and season of blood sampling, serum 25(OH)D was associated with lower odds of severe OSA [odds ratio (95% confidence interval): 0.94 (0.90–0.98)]. In the same multivariate model, VDD was associated with ~threefold higher odds of severe OSA [2.75 (1.38–5.48)]. In stratified analyses, VDD predicted OSA severity in the group of participants ≥50 y [3.54 (1.29–9.68)] and among those with body mass index ≥ 30 kg/m^2^ [3.38 (1.52–7.52)], but not in the younger and non-obese adults. This study provides further evidence of an inverse association between vitamin D levels and OSA severity and underscores the importance of considering vitamin D status as a potential modifiable factor in the comprehensive management of OSA.

## 1. Introduction

Obstructive sleep apnea (OSA) is a prevalent sleep-related breathing disorder, characterized by repetitive upper airway obstructions during sleep, leading to hypoxemia, hypercapnia, and frequent arousals [1]. Its increasing prevalence and association with various adverse health outcomes, notably cardiometabolic diseases, have garnered scientific attention [2]. Although OSA’s pathogenesis is not completely elucidated, it is widely accepted as multifactorial, involving mechanisms like insulin resistance, inflammation, and oxidative stress [3].

Vitamin D, a fat-soluble secosteroid, plays a crucial role in bone homeostasis regulation. While it is synthesized in the body upon exposure to ultraviolet light, it is also present in dietary sources, such as fatty fish, egg yolk, and fortified foods [4]. Nevertheless, vitamin D deficiency (VDD) is a common public health issue worldwide, even in Mediterranean countries with plentiful sunshine [5]. A continuous body of evidence relates low vitamin D levels with major health outcomes, including obesity, cardiovascular diseases, and diabetes mellitus. This is mainly due to the identification of vitamin D receptors in most body cells and tissues, which thus affect many bodily processes [6].

Mounting evidence suggests a bidirectional relationship between OSA and serum vitamin D levels. On the one hand, VDD may be involved in OSA pathogenesis. Meta-analyses of the available studies have shown that, compared to healthy controls, adults with OSA have lower serum 25-hydroxyvitamin [25(OH)] D levels, and VDD is exacerbated with increasing OSA severity [7]. It has also been reported that individuals with OSA have lower serum 25(OH)D levels and a higher VDD prevalence, independent of age or body mass index (BMI) [8]. On the other hand, OSA may contribute to VDD. It has been reported that vitamin D status is improved after long-term treatment with continuous positive airway pressure (CPAP) (i.e., the first-line treatment for OSA) [9]. In addition, adults with OSA are prone to having limited access to outdoor activities and diminished sunlight exposure due to daytime sleepiness, fatigue, or obesity, resulting in decreased synthesis of vitamin D [10].

However, the exact mechanism of VDD in adults with OSA and the correlation between vitamin D levels and OSA severity remain ambiguous, with contradictory results in clinical studies [11]. A common challenge is the variability in methodologies for assessing both vitamin D levels and OSA severity, hindering the generalizability of the findings. Moreover, other limitations lie in the limited consideration of confounding factors that could influence the relationship between vitamin D status and OSA severity. Notably, factors such as physical activity, smoking, and dietary habits, playing pivotal roles in both vitamin D metabolism and OSA, have not consistently been taken into account.

Given the existing discrepancies regarding the association between OSA and VDD and its potential clinical implications, the present study aimed to explore associations between serum 25(OH)D levels and polysomnographic parameters in a cohort of Greek adults with mild/moderate-to-severe OSA evaluated by polysomnography.

## 2. Materials and Methods

A detailed description of the study design, volunteer recruitment, and methodology is available in previously published reports from our study group [12,13]. Briefly, in this cross-sectional study, over a 4-year period (2014–2018), adults with suspected sleep-disordered breathing were evaluated at the Center of Sleep Disorders of Evangelismos Hospital and underwent overnight attended polysomnography (PSG). A detailed questionnaire collected information on volunteers’ sociodemographic characteristics, medical history, medication use, smoking habits, alcohol consumption, and attempts to change body weight/lifestyle. Exclusion criteria included other sleep disorders, major comorbidities, recent hospitalization/surgery, medication affecting sleep/body weight, alcohol abuse, and substantial alterations in body weight/lifestyle in the year preceding OSA diagnosis. Out of 357 adults diagnosed with OSA within the past 6 months, 88 were excluded for various reasons, leaving 269 enrolled after providing written informed consent. However, for the present analyses, the final sample consisted of 262 adults with available data on serum vitamin D status. The study protocol was approved by the Bioethics Committee of Harokopio University of Athens and by the Scientific Board of Evangelismos Hospital, in accordance with the Helsinki Declaration.

### 2.1. Assessment of Sleep Parameters

An overnight in-hospital PSG was conducted according to standard procedures [14], assessing brain activity, eye movements, muscle activity, heart rhythm, respiratory airflow, respiratory effort, and blood oxygen levels. A sole trained sleep technologist monitored and scored the recordings, which were saved on a multi-channel digital system. The American Academy of Sleep Medicine guidelines classify respiratory events as obstructive apneas and hypopneas, with the apnea–hypopnea Index (AHI) calculated as the number of events per hour of sleep. Participants were categorized by OSA severity: mild/moderate (AHI: 5–29 events/h) or severe (AHI ≥ 30 events/h) [15]. The apnea index (AI) was calculated as the number of obstructive apneas per hour of sleep, while the hypopnea index (HI) was defined as the number of hypopneas per hour of sleep. The oxygen desaturation index (ODI) was calculated as the number of respiratory episodes co-occurring with a decrease in blood oxygen saturation of ≥3% per hour of sleep. Wake time and time spent in non-rapid eye movement (NREM) and rapid eye movement (REM) sleep were also evaluated. Participants’ typical nighttime sleep duration (in hours) was categorized as short (≤6 h), adequate (7–9 h), or long (>9 h) [16]. The severity of sleep difficulty was determined using the Athens Insomnia Scale (AIS), which has a score range of 0 (no issues) to 24 (the most severe degree of insomnia) [17]. Finally, the Epworth Sleepiness Scale (ESS) was used to measure daytime sleepiness, with a maximum total score of 24 [18].

### 2.2. Anthropometric, Dietary, and Lifestyle Habits Assessment

Body weight, height, and waist circumference (WC) were measured, based on standard procedures [19]. BMI was calculated as weight (kg) divided by height squared (m^2^).

Dietary intake over the last 6 months before OSA diagnosis was assessed using a 76-item semi-quantitative food frequency questionnaire, including all food groups and individual foods/beverages commonly consumed in Greece [20]. Dietary intake was expressed in terms of food groups and individual foods and beverages [13]. Moreover, dietary intake was also assessed using three non-consecutive 24 h dietary recalls (2 weekdays and 1 weekend day). Recalls were analyzed in terms of energy and macronutrient intake using the Nutritionist ProTM software (Axxya Systems, Woodinville, WA, USA). Mean daily vitamin D intake (μg/d) was also extracted. Vitamin D and multivitamin supplementation use were also recorded.

Assessment of physical activity level was performed using the short version of the International Physical Activity Questionnaire [21], providing information on weekly time spent on walking, on sedentary activities, on moderate-intensity activities, and on vigorous-intensity activities during a usual week of the 6 months prior to PSG. The duration of daily physical activity (min/day) and the sum of weekly minutes of metabolic equivalent of tasks (METmin/week) were calculated [13].

### 2.3. Assessment of Vitamin D Status

Serum vitamin D status was evaluated by measuring the 25(OH)D levels, which is generally accepted as the most accurate indicator of overall body vitamin D status. Specifically, a direct, competitive chemiluminescence immunoassay was performed to measure 25(OH)D in ng/mL (LIAISON^®^ 25OH Vitamin D TOTAL Assay, Automated Analyzer Liaison Diasorin, ΜA-002/A.8/04-04-2019, DiaSorin Inc., Stillwater, MN, USA). In agreement with the Endocrine Society guidelines [22], serum 25(OH)D levels < 20 ng/mL were defined as VDD. Given the fact that serum 25(OH)D levels may vary depending on the season, the seasons of the participants’ blood draws were used as a confounder in the analyses (winter: December–February; spring: March–May; summer: June–August; autumn: September–November). 

### 2.4. Statistical Analysis

For the purpose of these secondary analyses, a post hoc test was performed, using the G-Power software, to calculate the exact power achieved by performing multiple logistic regression analysis, considering the effect size of serum vitamin D status on OSA severity and a type-I error rate of 0.05. The final sample achieved a power >90%.

The normality of the data was assessed using the Kolmogorov–Smirnov test. Continuous variables are presented as medians (interquartile range, IQR), while categorical variables are presented as frequencies. Correlations between vitamin D levels and sleep parameters were tested using Spearman’s correlation coefficient. The calculations of a chi-squared test or Mann–Whitney U test were used where appropriate to test differences between patients with VDD vs. those without. Multiple logistic regression analysis was applied to test the associations between vitamin D status, expressed as either 25(OH)D levels or VDD, and the presence of severe OSA, and the results are presented as odds ratios (OR) and their corresponding 95% confidence intervals (CI).

Confounding was evaluated using prior knowledge regarding biological relevance, as well as descriptive statistics from our study population. The covariates considered in full models included age, sex, smoking, BMI, physical activity level, dietary vitamin D intake, and season of blood sampling. A sensitivity analysis was also conducted among participants not taking vitamin D supplements (n = 241). Subgroup analyses based on age and BMI were also conducted to identify potential variations in the association between vitamin D status and OSA severity.

All statistical calculations were conducted using the Statistical Package for Social Sciences software (SPSS, version 21.0, Chicago, IL, USA) and all reported *p*-values < 0.05 (two-sided) were considered statistically significant.

## 3. Results

**Figure 1:**
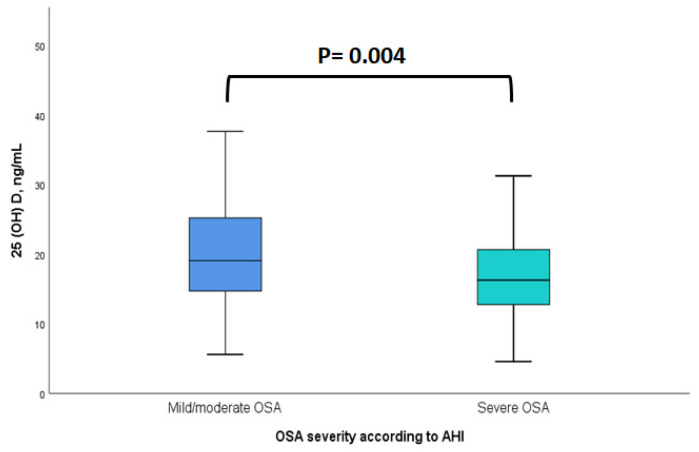
Serum 25(OH)D levels (median) by OSA severity according to AHI. Mild/moderate OSA: AHI 5 to <30 events/h; severe OSA: AHI ≥ 30 events/h. Abbreviations: 25(OH)D = 25-hydroxyvitamin D; OSA = obstructive sleep apnea; AHI = apnea–hypopnea index.

The descriptive characteristics of the participants are presented in Table 1. The sample consisted of 262 newly diagnosed adults with OSA, with a median age of 45 years (range 21–79 y) and a median (IQR) AHI of 47 (23–80) events/h. The majority of them were male (72.9%), obese (BMI ≥ 30 kg/m^2^) (77.1%), and had severe OSA (AHI ≥ 30 events/h) (67.2%). Participants reported low levels of physical activity (66.0% were engaged in physical activity for less than 30 min/d) and a median night-time sleep duration of 6 h/d, and 33.2% were current smokers. The median (IQR) dietary vitamin D intake was 1.70 (0.81, 3.26) μg/d, and only 8.0% of the participants reported taking dietary supplements containing vitamin D. Compared to adults with mild/moderate OSA, those with severe OSA exhibited lower serum 25(OH)D levels [median (IQR): 16.8 (13.1–21.7) vs. 19.2 (14.8–25.4) ng/mL *p* = 0.004] (Figure 1), and VDD was more prevalent among participants with severe OSA (68.2% vs. 52.4%, *p* = 0.011).

Table 1 also shows comparisons of various parameters between participants with and without VDD. Compared with OSA adults without VDD (n = 97), those with serum 25(OH)D levels < 20 ng/mL had higher median (IQR) values of AHI [54 (27–81) vs. 39 (18–74) events/h, *p* = 0.019] and lower values of lowest SpO_2_ [81 (72–85) vs. 83 (76–87) %, *p* = 0.019] and total sleep time (TST) [182 (140–305) vs. 231 (165–318) min, *p* = 0.037]. In addition, adults with VDD had higher BMI [35.8 (31.2–40.2) vs. 32.8 (29.0–36.1), *p* < 0.001] and WC values [males: 120 (109–132) vs. 114 (107–124) cm, *p* = 0.016; females: 115 (101–124) vs. 106 (88–112) cm, *p* = 0.004], and were more likely to experience sleep difficulties and insomnia, as expressed by AIS score [9 (5–13) vs. 7 (4–11), *p* = 0.026)]. No differences were observed according to VDD presence in other parameters (Table 1). 

Univariate analyses in the total sample showed that serum 25(OH)D levels were negatively correlated with BMI (rho = −0.289, *p* < 0.001), WC (rho = −0.221, *p* < 0.001), AHI (rho = −0.177, *p* = 0.004), ODI (rho = −0.137, *p* = 0.040), AHI in NREM sleep (rho = −0.144, *p* = 0.033), and AIS score (rho = −0.139, *p* = 0.025), whereas they were positively correlated with TST (rho = 0.163, *p* = 0.015), average SpO_2_ (rho = 0.152, *p* = 0.022), and lowest SpO_2_ (rho = 0.150, *p* = 0.017).

According to multiple logistic regression analysis (Table 2), each 1 ng/mL increase in serum 25(OH)D levels was associated with a 5% lower likelihood of severe OSA [OR (95% CI): 0.95 (0.92–0.98)] after adjusting for age, sex, and smoking (model 1). Further adjustments for BMI, physical activity level, and dietary vitamin D intake (model 2), as well as for season of blood sampling (model 3), revealed that the observed associations remained statistically significant (0.95 (0.91–0.99) and 0.94 (0.90–0.98), respectively). In similar models adjusted for the same confounders, VDD presence was associated with an approximately 2.7 times higher likelihood of severe OSA (OR (95% CI): 2.75 (1.38–5.48), in the fully adjusted model in Table 2). Sensitivity analyses among participants not receiving dietary supplements containing vitamin D (n = 241) yielded similar results.

Stratified analyses by BMI group (<30 vs. ≥30 kg/m^2^) and by age (<50 vs. ≥50 y) were also performed, and the results are shown in Table A1 and Table A2. Among obese participants with OSA (BMI ≥ 30 kg/m^2^), VDD was associated with ~3.4 times higher likelihood of severe OSA [OR (95% CI): 3.38 (1.52–7.52) in the fully adjusted model, Table A1], whereas VDD predicted OSA severity only in the group of participants ≥50 years [3.54 (1.29–9.68) in the fully adjusted model, Table A2]. No statistically significant associations were observed in the younger (<50 years) or non-obese participants (BMI < 30 kg/m²).

## 4. Discussion

The current cross-sectional study explores the associations between vitamin D status, polysomnographic parameters, and OSA severity, revealing significant correlations of serum 25(OH)D levels with polysomnographic indices. Furthermore, when accounting for several confounders in multivariate analyses, higher serum 25(OH)D levels were associated with a lower likelihood of severe OSA, while the presence of VDD was associated with a higher likelihood of severe OSA.

According to our results, a significant percentage (63%) of individuals with OSA exhibited inadequate levels of vitamin D. This finding aligns with previous studies in Greece showing a high prevalence of VDD in different population clusters [23,24]. Various risk factors, including obesity, advanced age, limited sunlight exposure, dark skin pigmentation, residential latitude, and socioeconomic status [25], may contribute to this high prevalence of VDD in the present cohort.

Serum 25(OH)D levels showed an adverse association with polysomnographic parameters, and a higher prevalence of VDD was observed among participants with severe OSA (AHI ≥ 30 events/h) compared to those with mild/moderate OSA. Serum 25(OH)D levels were also positively correlated with TST, corroborating previous studies indicating that individuals with VDD tend to have shorter sleep durations. This relationship suggests a potential bidirectional interaction between sleep patterns and vitamin D metabolism. Shortened sleep duration may disrupt circadian rhythms, affecting the synthesis and metabolism of vitamin D, while vitamin D deficiency could impact sleep quality and duration through mechanisms such as alterations in neurotransmitter pathways [26].

In addition, both serum 25(OH)D levels and the prevalence of VDD emerged as significant predictors of severe OSA. These observations are in accordance with previous studies showing that VDD is more prevalent in individuals with severe OSA [7,27]. Barcelo et al. [28] reported lower serum 25(OH)D in adults with severe OSA compared to those with milder forms of the disorder, whereas in a cohort of OSA patients with several comorbidities, Bouloukaki et al. [29] found that the presence of severe OSA was associated with a twofold-increased risk of VDD [OR (95% CI): 2.00 (1.05–3.82)]. In contrast with our findings, this association was significantly stronger in the younger (<60 y) and non-obese OSA patients.

The mechanisms explaining the relationship between vitamin D status and OSA are not completely elucidated. The association is likely to be bidirectional and multi-factorial. It is known that the repetitive pauses in breathing during sleep, which characterize OSA, lead to hypoxia, which may contribute to the activation of inflammatory pathways [30]. Inflammatory processes may impact the metabolism of vitamin D, potentially reducing its synthesis or availability. This hypothesis is further supported by the fact that higher adherence to CPAP treatment is associated with higher levels of 25(OH)D [31], and by recently published data from our research group showing that serum 25(OH)D levels are negatively correlated with plasma high-sensitivity C-reactive protein in the same cohort [32]. In addition, vitamin D levels were inversely correlated with sleep stage transitions, indicating the role of sleep fragmentation in VDD [11].

The relationship of vitamin D status with OSA severity can also be explained by obesity. OSA and VDD are both associated with obesity [1,33]. Notably, some individuals with obesity may exhibit a tendency to avoid outdoor activities, leading to insufficient sun exposure and, subsequently, reduced synthesis of vitamin D [34]. Furthermore, since vitamin D is fat-soluble, it tends to be stored in the adipose tissue. In the context of obesity, where there is an increased volume of adipose tissue, the storage distribution of vitamin D is elevated. This phenomenon results in a decreased release of vitamin D into the circulation, leading to lower bioavailability [35]. OSA exacerbates obesity, further contributing to sleep disturbances. Consequently, this vicious cycle leads to significantly lower vitamin D levels in individuals with obesity and OSA [7].

In our study, even after controlling for BMI in multivariate models, a significant correlation between VDD and severe OSA persisted. Stratified analyses by BMI showed that, among participants with BMI ≥ 30 kg/m^2^, VDD was associated with a ~3.4-times higher likelihood of severe OSA, highlighting that both OSA and obesity may further elevate the risk of low levels of vitamin D. A similar association of VDD with an increase in the risk of severe OSA was not evident in the group of non-obese adults in this study (BMI < 30 kg/m^2^); however, the study’s participants were mainly obese (77.1%), which may have limited the power to detect associations among non-obese adults. Interestingly, VDD predicted OSA severity in the group of older participants (≥50 y), but not in younger adults. OSA and VDD appear to have overlapping risk factors, including obesity and advancing age, and it is noteworthy that older adults typically exhibit lower vitamin D levels, as aging itself constitutes an independent risk factor for VDD [11].

On the other hand, low levels of vitamin D could contribute to the development of more severe OSA. Vitamin D plays a crucial role in muscle function and overall muscle health [36]. Skeletal muscle impairment is common in OSA, contributing to respiratory disturbances during sleep [37]. VDD may exacerbate muscle dysfunction, potentially impacting respiratory muscle strength and contributing to the severity of OSA [11]. It has been reported that a decline in the strength of the pharyngeal dilator muscles due to VDD may diminish pharyngeal patency, increasing susceptibility to apneic events during sleep [38].

The present study possesses several notable strengths. First, the utilization of attended in-hospital PSG for the diagnosis of OSA ensured a comprehensive and accurate assessment of sleep disordered breathing and allowed for the precise categorization of OSA severity. Secondly, the implementation of a standardized protocol for serum 25(OH)D assays is a key strength of our study. All measurements were conducted simultaneously using identical assays in the same laboratory, thereby minimizing measurement variability and enhancing the precision of vitamin D status assessments. This approach adds a layer of methodological consistency which is often lacking in other research. Thirdly, the exclusion of participants with additional major health conditions enhances the internal validity of our findings, allowing us to focus specifically on the association between vitamin D status and OSA severity without the confounding influence of comorbidities. Finally, our study benefitted from thorough confounding testing in statistical analyses by adequately considering factors such as physical activity, smoking, and dietary habits, which are known to be associated with both vitamin D status and OSA. 

The major limitation of the present study is its cross-sectional nature, restricting the ability to establish causation. Additionally, the reliance on questionnaires to assess participants’ lifestyle habits introduces the potential for recall bias. The study’s generalizability in other patients with OSA may also be limited, as our participants were drawn from a single clinical setting and had a specific profile (mainly middle-aged, obese men). Moreover, while we adjusted for various confounding factors, there might still be unaccounted variables influencing the observed associations.

## 5. Conclusions

The present study provides evidence of an inverse association between vitamin D levels and OSA severity, highlighting potential avenues for future research. While our findings are consistent with the existing literature, the complex interplay between vitamin D and OSA warrants further exploration through longitudinal studies and clinical trials. Understanding the underlying pathways linking VDD and OSA may open new therapeutic possibilities, including interventions aimed at optimizing vitamin D status to mitigate OSA severity. Our study, in conjunction with a growing body of evidence, underscores the importance of considering vitamin D status as a potential modifiable factor in the comprehensive management of OSA.

## Figures and Tables

**Table 1:** General characteristics and sleep parameters of 262 adults with obstructive sleep apnea in total and according to the presence of 25(OH)D deficiency.

	Total Sample	with 25(OH)D <20 ng/mL	with 25(OH)D ≥20 ng/mL	*p* ^a^
N (%)	262 (100)	165 (63)	97 (37)	
Age (y)	49 (41–56)	48 (41–54)	50 (43–58)	0.032
Males, n (%)	191 (72.9)	114 (69.1)	77 (79.4)	0.047
BMI, kg/m^2^	34.3 (30.4–39.1)	35.8 (31.2–40.2)	32.8 (29.0–36.1)	<0.001
BMI ≥ 30 kg/m^2^	202 (77.1)	134 (81.2)	68 (70.1)	0.029
WC, cm				
Males	116 (108–128)	120 (109–132)	114 (107–124)	0.016
Females	110 (96–120)	115 (101–124)	106 (88–112)	0.004
Increased WC, n (%) ^b^	216 (82.4)	140 (90.9)	76 (79.2)	0.008
Current smokers, n (%)	87 (33.2)	49 (29.7)	38 (39.2)	0.076
Socioeconomic status, n (%) ^c^				0.309
Low	71 (28.2)	43 (26.9)	28 (30.4)	
Moderate	139 (55.2)	86 (53.8)	53 (57.6)	
High	42 (16.7)	31 (19.4)	11 (12.0)	
Physical activity, METmin/week	347 (100-990)	297 (83–908)	524 (99–991)	0.214
Physical activity ≥ 30 min/d	89 (34.0)	51 (30.9)	38 (39.2)	0.180
Total energy intake, kcal/d	2945 (2553–3372)	2898 (2539–3357)	2989 (2569–3475)	0.360
Vitamin D intake, μg/d	1.70 (0.81-3.26)	1.66 (0.77–3.47)	1.76 (0.88–3.14)	0.826
Use of vitamin D containing supplements, n (%)	21 (8.0)	9 (5.5)	12 (12.4)	0.059
Sleep parameters				
TST (minutes)	197 (152–312)	182 (140–305)	231 (165–318)	0.037
N1 (%TST)	3.4 (1.5–7.8)	3.6 (1.4–6.6)	3.0 (1.5–6.7)	0.379
N2 (%TST)	79.5 (68.9–88.4)	79.6 (68.1–86.8)	79.5 (70.3–89.6)	0.483
N3 (%TST)	0 (0.0–8.8)	0 (0.0–8.8)	0 (0.0–9.3)	0.854
REM (%TST)	11.3 (2.8–16.9)	11 (3.5–15.7)	11.9 (2.7–19.2)	0.396
AHI (events/h)	47 (23–80)	54 (27–81)	39 (18–74)	0.019
AHI ≥ 30, n (%)	176 (67.2)	120 (73.6)	56 (58.9)	0.011
AI (events/h)	23 (8–60)	24 (8–61)	22 (8–60)	0.603
HI (events/h)	11 (5–20)	11 (5–20)	9 (5–21)	0.476
NREM AHI (events/h)	44 (21–81)	52 (23–82)	41 (17–76)	0.123
REM AHI (events/h)	54 (26–79)	59 (33–79)	42 (19–79)	0.115
ODI (events/h)	37 (14–66)	39 (18–67)	33 (10–65)	0.083
Average SpO_2_ (%)	93 (91–94)	93 (90–94)	93 (91–95)	0.076
Lowest SpO_2_ (%)	82 (74–86)	81 (72–85)	83 (76–87)	0.019
Mean pulse, beats/min	69 (63–77)	71 (65–78)	67 (60–73)	0.003
Sleep efficiency (%)	90.6 (80.0–97.2)	89.1 (80.1–97.5)	90.9 (79.0–96.6)	0.798
Sleep latency, min	43 (19–82)	41 (15–79)	47 (22–93)	0.083
Self-reported sleep duration, h/d	6 (5–7)	6 (5–7)	6 (5–7)	0.977
AIS score (0–24)	8 (5–12)	9 (5–13)	7 (4–11)	0.026
ESS score (0–24)	10 (7–14)	11 (8–14)	10 (6–14)	0.413

Data are presented as medians (IQR) for numerical variables and as absolute numbers (relative frequency) for categorical variables. ^a^ Differences in variables were tested using the Mann–Whitney U test for continuous variables and a chi-squared test for categorical variables. ^b^ Males: >102 cm, females: >88 cm. ^c^ Participants were classified into 3 socioeconomic status (SES) categories: (i) low SES (i.e., up to 9 years of education and low/medium income (<EUR 20,000/year), or up to 14 years of education and low income (<EUR 10,000/year)); (ii) high SES (i.e., 10 or more years of education and high income (>EUR 20,000/year)); and (iii) moderate SES, including the rest of the participants. Abbreviations: OSA = obstructive sleep apnea; 25(OH)D = 25-hydroxyvitamin D; BMI = body mass index; WC = waist circumference; MET = metabolic equivalent of task; TST = total sleep time; REM = rapid eye movement; NREM = non-rapid eye movement; AHI = apnea–hypopnea index; AI = apnea index; HI = hypopnea index; ODI = oxygen desaturation index; SpO_2_ = blood oxygen saturation; AIS = Athens insomnia scale; ESS = Epworth sleepiness scale.

**Table 2:** Logistic regression analysis models exploring the association between serum 25(OH)D status and the likelihood of severe OSA (AHI ≥ 30 events/h) (n = 262).

	Model 1		Model 2		Model 3	
	OR	95% CI	OR	95% CI	OR	95% CI
25(OH)D, ng/mL	0.949	0.915–0.984	0.949	0.909–0.990	0.938	0.896–0.983
25(OH)D < 20 ng/mL	2.723	1.486–4.987	2.482	1.276–4.830	2.751	1.381–5.483

Model 1: adjusted for age, sex, and smoking. Model 2: Model 1 + adjusted for BMI, physical activity level, and dietary vitamin D intake. Model 3: Model 2 + adjusted for season of blood sampling. Abbreviations: 25(OH)D = 25-hydroxyvitamin D; OSA = obstructive sleep apnea; AHI = apnea–hypopnea index; OR = odds ratio; CI = confidence interval; BMI = body mass index.

## Data Availability

Deidentified participant data and the study protocol will be made available by the corresponding author upon request.

## Appendix A

**Table A1:** Associations between serum 25(OH) D status and the likelihood of severe OSA (AHI ≥ 30 events/h) by BMI group.

	BMI < 30 kg/m^2^		BMI ≥ 30 kg/m^2^	
	OR	95% CI	OR	95% CI
25(OH)D < 20 ng/mL				
Model 1	1.64	0.51–5.25	2.98	1.41–6.31
Model 2	2.11	0.59–7.61	3.13	1.43–6.84
Model 3	2.09	0.53–8.31	3.38	1.52–7.52

Model 1: adjusted for age, sex, and smoking. Model 2: Model 1 + adjusted for physical activity level and dietary vitamin D intake. Model 3: Model 2 + adjusted for season of blood sampling. Abbreviations: 25(OH)D = 25-hydroxyvitamin D; OSA = obstructive sleep apnea; AHI = apnea–hypopnea index; OR = odds ratio; CI = confidence interval; BMI = body mass index.

**Table A2:** Associations between serum 25(OH) D status and the likelihood of severe OSA (AHI ≥ 30 events/h) by age group.

	Age < 50 y		Age ≥ 50 y	
	OR	95% CI	OR	95% CI
25(OH)D < 20 ng/mL				
Model 1	2.26	0.97–5.28	2.89	1.25–6.64
Model 2	2.08	0.78–5.55	2.91	1.13–7.51
Model 3	2.25	0.79–6.42	3.54	1.29–9.68

Model 1: adjusted for sex and smoking. Model 2: Model 1 + adjusted for BMI, physical activity level, and dietary vitamin D intake. Model 3: Model 2 + adjusted for season of blood sampling. Abbreviations: 25(OH)D = 25-hydroxyvitamin D; OSA = obstructive sleep apnea; AHI = apnea–hypopnea index; OR = odds ratio; CI = confidence interval; BMI = body mass index.
